# Agent-Based Modeling for Super-Spreading Events: A Case Study of MERS-CoV Transmission Dynamics in the Republic of Korea

**DOI:** 10.3390/ijerph15112369

**Published:** 2018-10-26

**Authors:** Yunhwan Kim, Hohyung Ryu, Sunmi Lee

**Affiliations:** 1 Division of Media Communication, Hankuk University of Foreign Studies, Seoul 02450, Korea; yunhwankim2@gmail.com; 2 Department of Applied Mathematics, Kyung Hee University, Yongin 446-701, Korea; kyinr108@gmail.com; 3 Institute of Natural Sciences, Kyung Hee University, Yongin 446-701, Korea

**Keywords:** 2015 MERS-CoV, super-spreading events, agent-based models, basic reproduction number, isolation interventions

## Abstract

Super-spreading events have been observed in the transmission dynamics of many infectious diseases. The 2015 MERS-CoV outbreak in the Republic of Korea has also shown super-spreading events with a significantly high level of heterogeneity in generating secondary cases. It becomes critical to understand the mechanism for this high level of heterogeneity to develop effective intervention strategies and preventive plans for future emerging infectious diseases. In this regard, agent-based modeling is a useful tool for incorporating individual heterogeneity into the epidemic model. In the present work, a stochastic agent-based framework is developed in order to understand the underlying mechanism of heterogeneity. Clinical (i.e., an infectivity level) and social or environmental (i.e., a contact level) heterogeneity are modeled. These factors are incorporated in the transmission rate functions under assumptions that super-spreaders have stronger transmission and/or higher links. Our agent-based model has employed real MERS-CoV epidemic features based on the 2015 MERS-CoV epidemiological data. Monte Carlo simulations are carried out under various epidemic scenarios. Our findings highlight the roles of super-spreaders in a high level of heterogeneity, underscoring that the number of contacts combined with a higher level of infectivity are the most critical factors for substantial heterogeneity in generating secondary cases of the 2015 MERS-CoV transmission.

## 1. Introduction

The 2015 outbreak of Middle East Respiratory Syndrome coronavirus (MERS-CoV) posed a serious public health threat in the Republic of Korea. It generated 186 confirmed cases including 38 fatal cases [1]. It has drawn a lot of attention not only because it was the largest outbreak of MERS-CoV outside the Middle East regions but also because it has been claimed to be a super-spreading event (SSE) similar to the SARS outbreaks in 2003 [2]. SSE is an outbreak where a small number of infected cases generated a large number of secondary infections [3]; for example, 20% of infectious individuals are responsible for 80% of newly generated infections. Super-spreading events have been observed in the transmission dynamics of many infectious diseases including SARS transmission in Hong Kong and Singapore [4]. Stein identified the complex interplay among hosts, pathogens and environments for super-spreading events in many infectious diseases [5]. Furthermore, Shen et al. defined SSE as a patient transmitting a disease to at least eight contacts, based on an analysis of the SARS spread data [6].

Concerning the 2015 MERS-CoV outbreak in Korea, previous studies have examined whether it was an SSE. Kucharski and Althaus analyzed a large transmission cluster of MERS-CoV in Korea to estimate the level of transmission heterogeneity and identified a substantial potential for super-spreading events [7]. Also, Chun identified that MERS-CoV transmission in Korea was an SSE using a negative binomial model [8]. Kim et al. characterized the transmission dynamics based on a mathematical model and investigated the time-dependent reproduction number [9]. Gerardo et al. performed the comparative analysis between SARS and MERS; they claimed that MERS-CoV in Korea has shown the most significant heterogeneity in generating secondary cases [2]. Nishiura et al. suggested that multiple visits to health facilities were the main cause of the disproportionate number of secondary cases and this might lead the outbreak to be an SSE [10].

**Figure 1:**
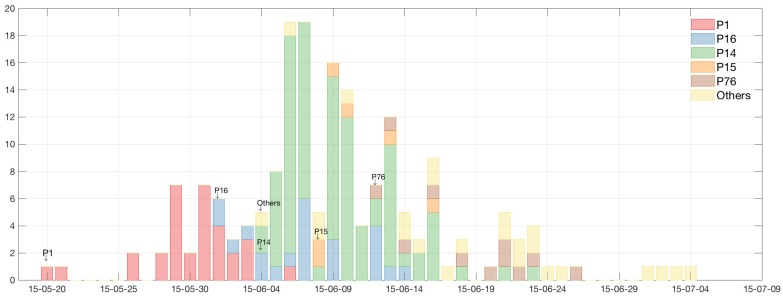
Time series of 2015 MERS-CoV cases is displayed and classified by five super-spreaders: these five colors represent the cases by each super-spreader (yellow color is unknown due to multiple contacts). At the beginning of the epidemic, the red bar indicates secondary cases by the index case ($P_{1}$). This shows significant heterogeneity in the 2015 MERS-CoV transmission: five super-spreaders (3%) infected 79% (147/186) of the total MERS-CoV cases.

**Figure 2:**
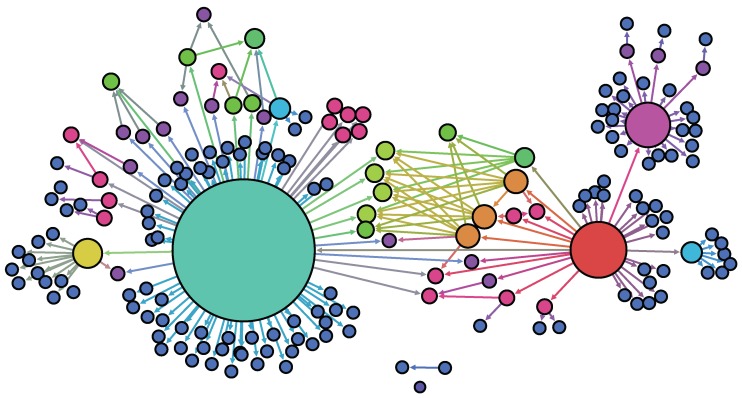
Transmission trees are displayed (only infection tracing shown). The node size is proportional to the number of secondary infection cases (five super-spreaders are identified).

The high level of heterogeneity in generating secondary cases, which is one of the key evidence of an SSE, can be observed in the time series of cases in 2015 MERS-CoV outbreak in Korea. In Figure 1, the time series of the 2015 MERS-CoV cases is displayed and classified using five super-spreaders: the five colors represent the different cases according to super-spreader (yellow color is unknown due to multiple contacts). It is clearly seen that there was significant heterogeneity in the 2015 MERS-CoV transmission: five super-spreaders (3%) infected 79% (147/186) of the total MERS-CoV cases. Transmission trees (infection tracing only) for the total 186 MERS-CoV cases are displayed in Figure 2. The node size is proportional to the number of secondary cases (five super-spreaders are identified as in Figure 1).

Having identified the MERS-CoV outbreak in Korea as an SSE, it is necessary to develop a mathematical model that can capture the heterogeneity of infectivity among patients. There have been various approaches to model the SSE in infectious disease transmission dynamics. Firstly, the standard compartmental models have been used. Mkhatshwa and Mummert developed a SIRP model by adding a new superspreader class to a standard SIR model [11]. They assumed that superspreaders have a longer infectious period than normal infected individuals. While their model was simple and clear, a SIR-type model is inherently limited in capturing the individual heterogeneity of attributes, including contact numbers and infectivity. Another way of modeling SSE is a branching process model. In each discrete time step, an infected individual produces a random number of secondary infections that follows a certain probability distribution. There are a small number of cases whose value is far from the mean and these are used to model super-spreaders. Lloyd-Smith et al. employed a negative binomial distribution and defined an SSE where any single patient infects more than the *n*th percentile of the distribution [12]. James, Pitchford and Plank suggested three branching process models, each of whose random variable follows a different probability distribution [13]. Garske and Rhodes suggested a branching process model implemented in continuous time instead of discrete generation [14].

In addition to these methods, agent-based modeling (ABM) can be a useful tool in modeling SSEs. Due to the dramatic increase in computing power and its strength in modeling individual heterogeneity, it has been employed in modeling various aspects of infectious disease transmission dynamics especially in modeling SSEs with spatio-temporal factors. In ABM, SSEs are usually modeled by incorporating a small number of agents (super-spreaders) who can generate a larger number of secondary infections than normal agents. There are a few ways to implement these super-spreaders. The first way is to make them have an inherently higher level of infectivity [14,15]: this corresponds to the higher transmission rate in a mathematical model. Another way is to make the infectious period of super-spreaders longer while the infectivity is same with normal agents [11,14]. The last way is to make the number of contacts of super-spreader larger than the number of normal agents while the infectivity remains same [16,17]. Each of these ways pays attention to different aspects of the super-spreaders and can be useful for capturing and analyzing SSEs.

In particular, a group of studies has employed ABM in modeling SSEs from the perspective of the contact network. Fujie and Odagaki developed agent-based models on an irregular lattice network and suggested two kinds of models in their work; a strong infectiousness model and a hub model [15]. Comparing the results of two models with SARS transmission data in Hong Kong, they concluded that the hub model provides a closer match with the real-world data. Small, Tse and Walker suggested a small-world network model where individuals have local links that connect to immediate neighbors and non-local links that connect to far-away nodes [16]. They represented the former as contact with the family and the latter as contact made in public spaces. Bifolchi, Deardon and Feng simulated the epidemic spreads through various contact networks to determine which model best predicts the true probabilities of infection [17]. Duan et al. suggested an elaborate agent-based model that incorporates a variety of features of agent behaviors that might generate SSE including heterogeneous levels of infectivity and contacts [18].

The present research aims to develop stochastic agent-based models of the 2015 MERS-CoV transmission dynamics in South Korea. Embracing the power of ABM, heterogeneities among agents are implemented in the models. In particular, regular-spreaders and super-spreaders are set to have different transmission rate functions based on different transmission probabilities and a different number of contacts. Based on these heterogeneities, four models are suggested: no super-spreader model (No-SS), the super-spreaders model with higher infectivity (SSM1), the super-spreaders model with higher contacts (SSM2), and super-spreaders model with both higher infectivity and higher contacts (SSM3). Monte Carlo simulations are carried out under various epidemic scenarios and the results are compared with the real epidemic data of 2015 MERS-CoV in Korea. The distributions of the basic reproduction number and epidemic outputs are computed as well as the distribution of secondary cases under different models of super-spreaders. Furthermore, the effectiveness of isolation intervention strategies and their impact on the MERS-CoV transmission dynamics are investigated.

## 2. Methods

### 2.1. SEIR Stochastic Agent-Based Model

**Figure 3:**
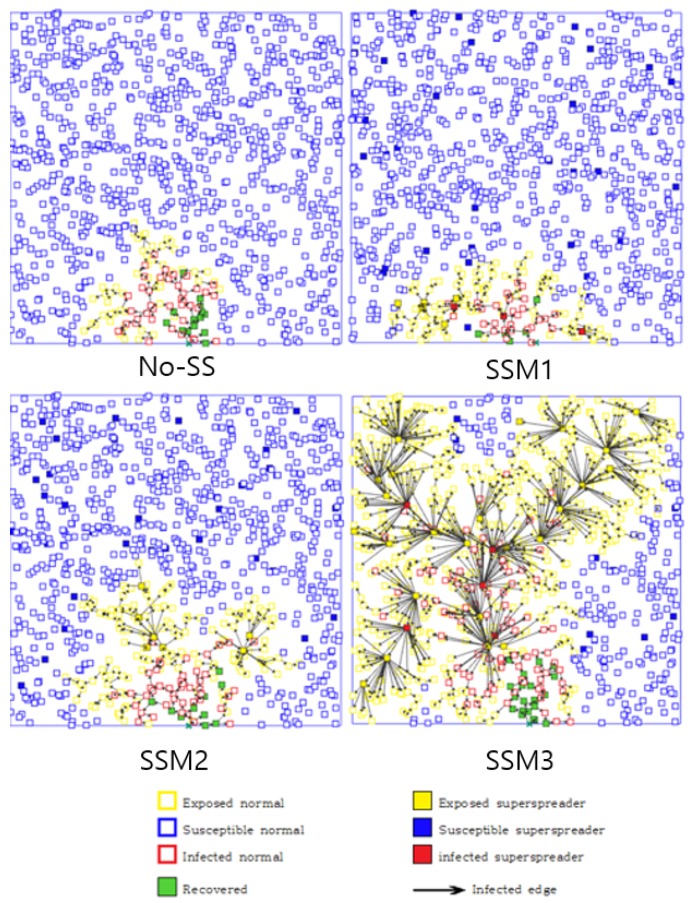

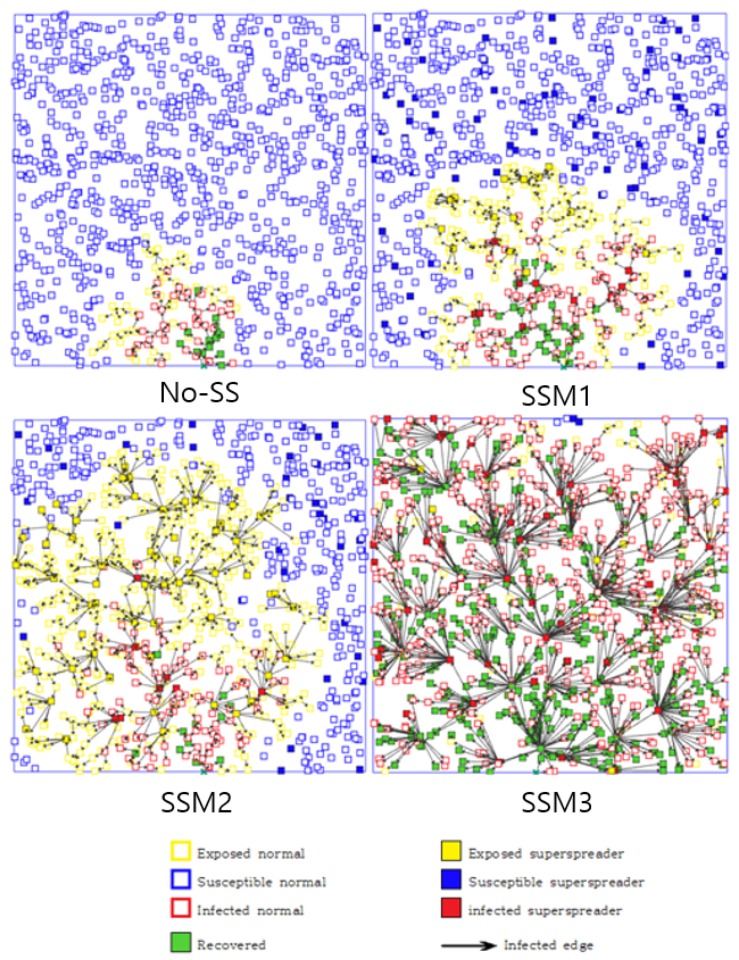
Transmission dynamics of four model are demonstrated at t=25 day (super-spreaders are indicated by a filled box). Top four panels show the results using λ=4% and bottom four panels show the results using λ=10%. SSM 3 shows the fastest transmission pattern while No-SS shows the slowest (the difference becomes more significant as λ increases).

We develop a stochastic agent-based model by generating a random network on a square domain L×L with a total number of agents *N* (as shown in Figure 3). Each agent can have one of the following four epidemiological statuses: Susceptible (S), Exposed (E), Infected (I), and Recovered (R). A susceptible agent becomes exposed with a probability defined by the transmission probability distribution, which will be descried below. We assume that a newly infected person becomes infectious (asymptomatic) and remains in the exposed stage for a incubation time drawn from a gamma probability density function (PDF) with a mean of 1/κ days and a standard deviation of $\sigma_{\kappa }$ days. After this time, the individual becomes infectious (symptomatic) and remains so for a duration of time drawn from a gamma PDF with a mean of 1/γ days and a standard deviation of $\sigma_{\gamma }$ days. Subsequently, an infected agent recovers with immunity. For simplicity, a gamma PDF is used for both the incubation and infectious period; these have been estimated from the 186 MERS-CoV confirmed cases provided by KCDC (2016) [1].

### 2.2. Transmission Rate Functions

Each agent is either a regular agent or a super-spreader, and the proportion of super-spreaders in the total population (*N* agents) is determined by the parameter λ. Agents in the *E* or *I* status can infect other S agents within a distance $r_{0}$ with the transmission probability function of β(r). Here, the distance between two agents located at $\left(x_{1},y_{1}\right)$ and $\left(x_{2},y_{2}\right)$ is calculated as $r=\sqrt{{\left(x_{2}-x_{1}\right)}^{2}+{\left(y_{2}-y_{1}\right)}^{2}}$. We define two types of transmission rate functions for regular-spreaders and super-spreaders. Here, super-spreaders are assumed to have either a higher transmission probability or larger number of contacts than regular spreaders (three different models for super-spreaders will be described below).

Let us define transmission rate functions for *regular-spreaders* and *super-spreaders*. The transmission rate function is defined as a function of the distance between exposed (or infected) and susceptible individuals.

- **Regular-spreader**:
  (1) $$\beta \left(r\right)=\left\{\begin{array}{c} \beta_{0}{\left(1-\frac{r}{r_{0}}\right)}^{\alpha },\;0\leq r\leq r_{0}\\ 0,\;r_{0}\leq r \end{array}\right.$$
  Here, β(r) is a decreasing function of the distance *r* within an effective contact radius $r_{0}$, and α is the exponent of the given function of distance *r* with α>1. Please note that this model is referred as to the no super-spreader model **No-SS** when there is no super-spreader agent (λ=0).
- **Super-Spreader Model 1**:
  (2) $$\beta_{1}\left(r\right)=\left\{\begin{array}{c} \beta_{0},\;0\leq r\leq r_{0}\\ 0,\;r_{0}\leq r \end{array}\right.$$
  Our first super-spreader model (**SSM1**) assumes that super-spreaders have a higher transmission probability (or higher infectivity) than regular spreaders within the effective contact radius $r_{0}$. Please note that $\beta_{1}\left(r\right)$ is a constant function remaining at a value of $\beta_{0}$ for $0\leq r\leq r_{0}$.
- **Super-Spreader Model 2**:
  (3) $$\beta_{2}\left(r\right)=\left\{\begin{array}{c} \beta_{0}{\left(1-\frac{r}{r_{n}}\right)}^{\alpha },\;0\leq r\leq r_{n}\\ 0,\;r_{n}\leq r \end{array}\right.$$
  The second super-spreader model (**SSM2**) assumes that super-spreaders have more links (or a larger number of contacts) than regular spreaders and is modeled by a longer effective contact radius with $r_{n}=\sqrt{6}r_{0}$.
- **Super-Spreader Model 3**:
  (4) $$\beta_{3}\left(r\right)=\left\{\begin{array}{c} \beta_{0},\;0\leq r\leq r_{0}\\ \beta_{0}{\left(1-\frac{r-1}{r_{n}-r_{0}}\right)}^{\alpha },\;r_{0}\leq r\leq r_{n} \end{array}\right.$$
  Lastly, we propose a hybrid model (**SSM3**) for super-spreaders by considering a combined transmission probability of SSM1 and SSM2 since super-spreaders have both a higher transmission probability and a larger number of contacts [1].

## 3. Simulation Results

All susceptible agents (either regular or super-spreaders) are located on an L×L space, and their positions are randomly assigned at the beginning and fixed during the entire simulation (see Figure 3). One infected agent (*the index case*) is randomly located on the bottom of the simulated space (all other agents are S status). A Monte Carlo simulation is carried out with 1000 trials using the same set of parameter values. The averaged output of 1000 runs (or distributions) is presented from our numerical simulations. Baseline parameter values are given in Table 1.

### 3.1. The Impact of Super-Spreaders on Epidemic Outputs

**Figure 4:**
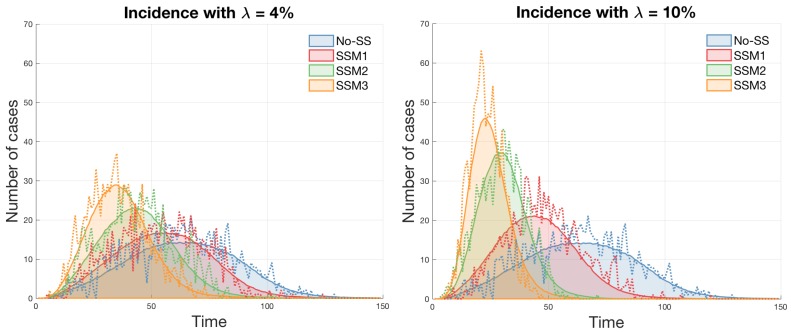
Incidence profiles are compared under four different models (left with λ=4% and right with λ=10%). The average incidence of 1000 simulations is displayed (a solid smooth curve) with a particular incidence (a dashed stochastic curve).

The impact of different super-spreading models is investigated in terms of various epidemic outputs, including incidence, peak size, peak time and epidemic duration. First, Figure 4 compares incidence profiles using two values of λ (a super-spreader proportion): left with λ=4% and right with λ=10%. The average incidence of 1000 simulations is displayed as a solid smooth curve along with one particular incidence (a dashed stochastic curve).

Clearly, the effect of super-spreaders makes the disease transmission faster and more severe (see SSM3 in both panels) since super-spreaders in SSM3 have a stronger transmission and longer effective radius than the other models (in order of SSM3 > SSM2 > SSM1 > No-SS). This becomes more profound under a higher proportion of super-spreaders in the right panel (λ=10%).

**Figure 5:**
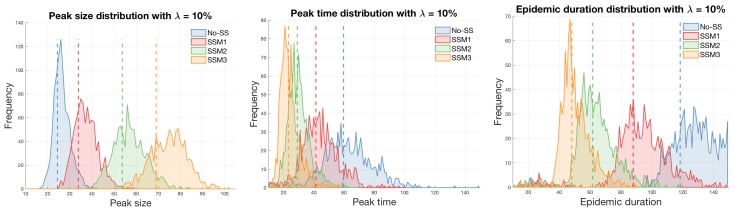
Distributions of three epidemic outcomes (peak size, peak time, and epidemic duration) are compared for each model using λ=10% (the mean of each epidemic output is displayed as a dashed line).

Next, the detailed epidemic distributions are illustrated in Figure 5, which shows the peak size, peak time, and epidemic duration using λ=10% with the mean represented by the dashed vertical line. The peak time is earlier and the peak size is larger in SSM3 (again in order of SSM3 > SSM2 > SSM1 > No-SS). This confirms that the impact of super-spreaders is significant on the MERS-CoV transmission dynamics. Also, the detailed epidemic outputs using two values of λ=4% and λ=10% are summarized in Table 2. Table 2 illustrates the mean and standard deviation under four models for three epidemic outputs (peak size, peak time, and epidemic duration).

Higher peak size, faster peak time, and earlier epidemic durations are the common features of SSE, which is consistent with the results under SSM3 as shown in the above epidemic outputs. This is due to the larger number of contacts and higher transmission rates resulting in the larger epidemic peak in a shorter period of time.

### 3.2. The Impact of Super-Spreaders on the Basic Reproduction Number

**Figure 6:**
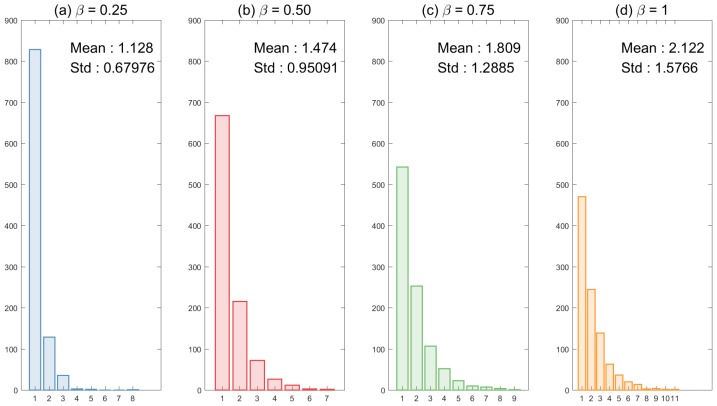

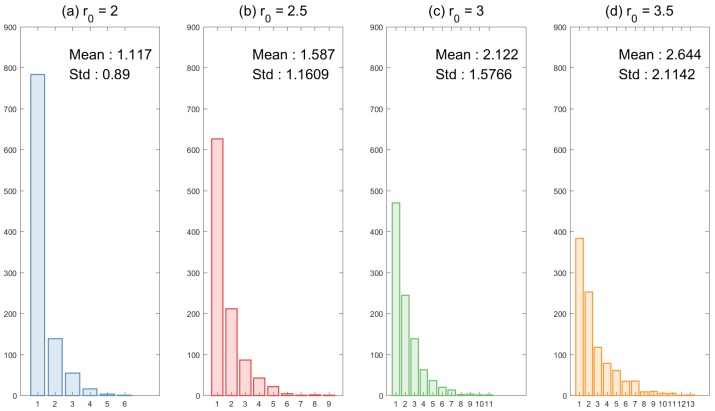
Sensitivity analyses for the distributions of $R_{0}$ are shown under different epidemic parameters: the top panels show the impact of the baseline transmission rate $\beta_{0}$ and the bottom panels show the impact of the effective radius $r_{0}$ (these results are under No-SS).

The basic reproduction number is one of the most important quantities in mathematical epidemiology. It defines the average number of secondary infections in a completely susceptible population. One can obtain an analytic expression of the basic reproduction number ($R_{0}$) for some compartment models [19]. However, in general, there is no analytic expressions of $R_{0}$ for agent-based models, hence, we employ the method for determining $R_{0}$ described in [20]. We compute the basic reproduction number from a randomly chosen infected individual in a completely susceptible population and obtain the distributions of 1000 simulations of secondary cases. Figure 6 shows the distributions of $R_{0}$ in the absence of super-spreaders (No-SS) and the mean is the dashed vertical line. Sensitivity analyses of $R_{0}$ are conducted by varying baseline transmission rates and an effective radius. In our simulation, the effective radius has the most significant impact and higher transmission rates lead to larger $R_{0}$.

**Figure 7:**
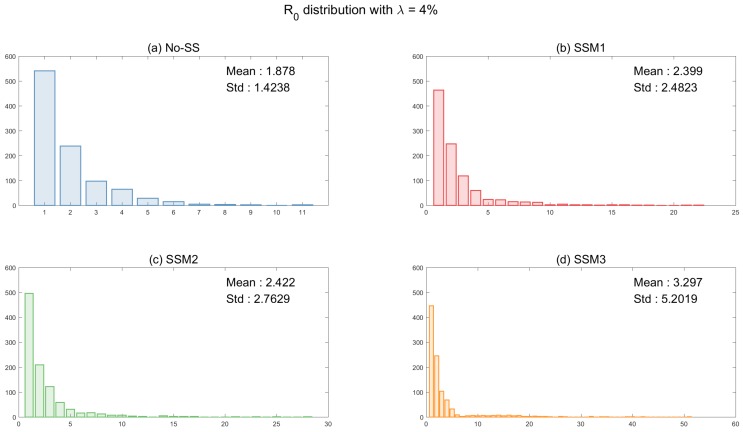

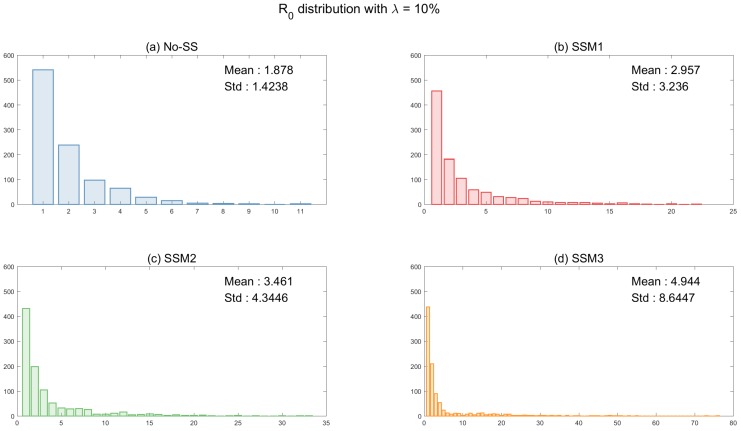
The distributions of $R_{0}$ are shown using two different proportion of super-spreaders. Distributions of $R_{0}$ under four models are compared (top with λ=4% and bottom with λ=10%).

Next, Figure 7 illustrates the distributions of $R_{0}$ under four super-spreader models (using four different transmission rate functions). It shows the impact of super-spreaders on $R_{0}$ using two values of λ (super-spreader proportion): on the top with λ=4% and the bottom with λ=10%. Since the basic reproduction number is computed only the first generation by the index case, the differences for $R_{0}$ distributions of four models are smaller when λ=4%. However, the impact of super-spreaders in SSM3 becomes more significant when λ=10%, with the maximum mean around 5 of SSM3 (the means are in order of SSM3 > SSM2 > SSM1 > No-SS in the bottom panels). Please note that there exists a larger right tail in SSM3 (in order of SSM3 > SSM2 > SSM1 > No-SS). Obviously, this implies that there is a higher probability for a larger number of secondary cases to appear under SSM3.

### 3.3. Further Sensitivity Analysis

**Figure 8:**
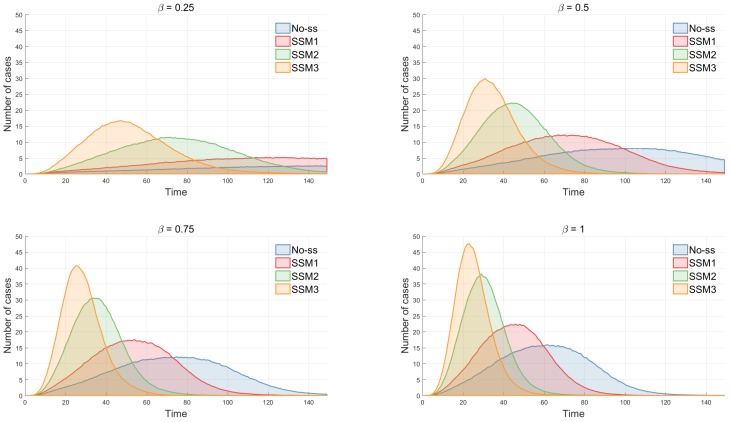

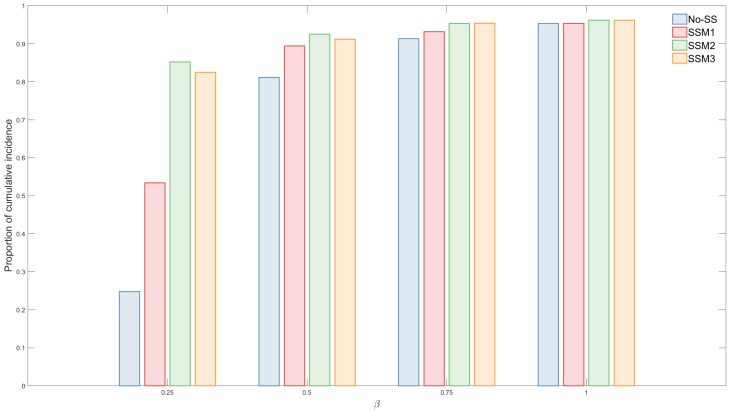
The impact of the baseline transmission rate $\beta_{0}$ is shown under four models. In all models, the peak size increases rapidly as $\beta_{0}$ increases. The difference is most significant in SSM 3.

**Figure 9:**
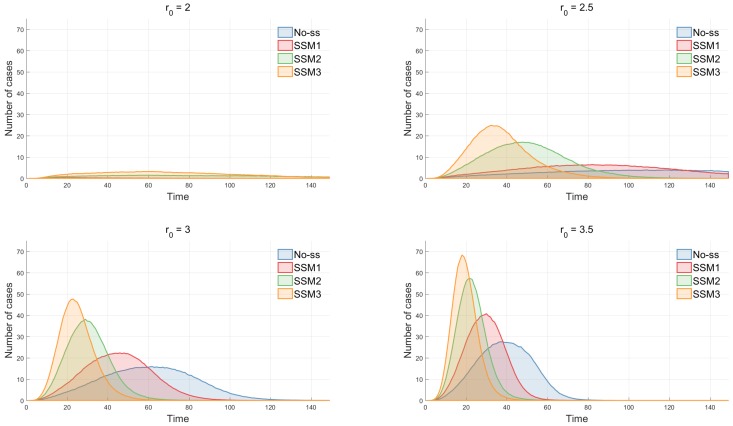

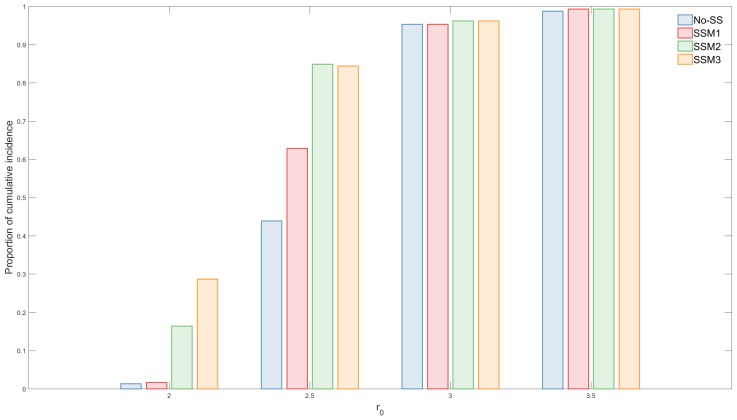
The impact of the effective radius $r_{0}$ is shown under four models. In all models, the peak size increases rapidly as $r_{0}$ increases. The difference is most significant in SSM 3. The change of proportion of cumulative incidence while varying the different values of $r_{0}$ is shown that, in each model, the outbreak probability increases rapidly as $r_{0}$ reaches a certain range of values.

The impact of three epidemic parameters is determined through incidence curves. First, the effect of the baseline transmission rate ($\beta_{0}$) on incidence has been investigated in Figure 8. In general, disease transmission becomes faster and stronger as $\beta_{0}$ increases; the peak time is earlier and the peak height is larger in all four models. Next, the impact of an effective contact radius ($r_{0}$) has been explored in Figure 9. There is a critical value of $r_{0}$ that determines the outbreak as pointed out in [15]. There is no outbreak when $r_{0}\leq 2$ and outbreaks occur when $r_{0}>2$. When $r_{0}$ reaches around 2.5, outbreaks take place in SSM2 and SSM3 and they get stronger in size and larger in speed as $r_{0}$ gets larger. It is straightforward that if patients can infect other individuals at a further distance, then disease will be transmitted faster. It accelerates the change of proportion of cumulative incidence by the different values of $r_{0}$.

Also, the proportion of super-spreaders (λ) is varied and the incidence profiles were compared (results are not shown). As the proportion of super-spreaders gets larger, the peak size becomes larger and the peak time earlier in SSM2 and SSM3, while it does not have a remarkable impact in SSM1. This suggests that a longer effective radius makes the disease spread easier. Lastly, we investigated the effect of the exponent term α on the transmission rate for super-spreaders. We vary the value of α (the exponent term in β(r)) for SSM1, SSM2 and SSM3. The transmission rate function decreases rapidly as α increases (results are not shown here). This leads to the strength and the speed of disease transmission decreasing as α increases. More details for these results can be found in [21].

### 3.4. The Impact of Super-Spreaders on Distributions of Secondary Cases

**Figure 10:**
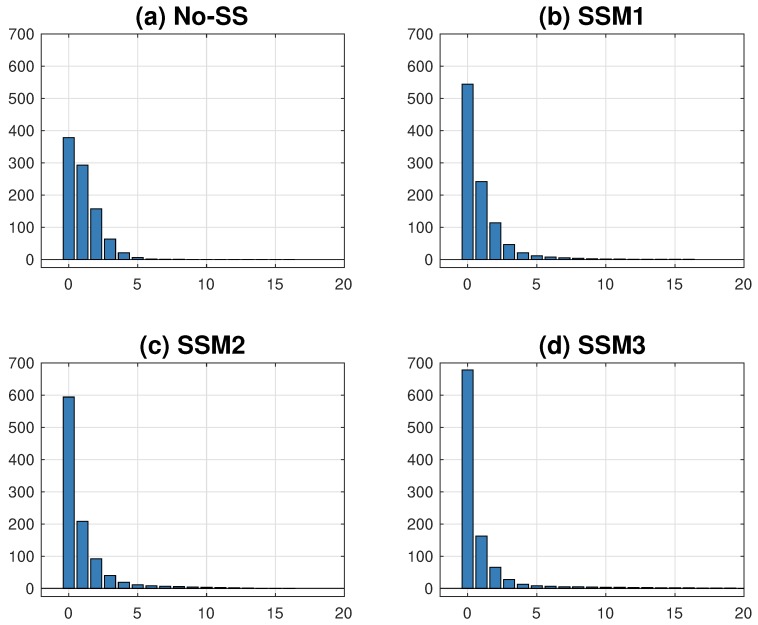
Distributions of secondary cases under four super-spreader models are compared using λ=10%. SSM3 shows the highest level of heterogeneity while No-SS shows the least heterogeneity.

As observed in many infectious diseases, we explore the distributions of secondary cases under four models. Again, 1000 simulations for each model have been carried out and the mean distributions are obtained. Figure 10 illustrates the mean distribution of the number of links under each model. In the absence of super-spreaders, No-SS shows monotone decreases in the number of links (the most homogeneous in four model outputs with the shortest tail from zero to six) (a). As the transmission rates become stronger and the number of contacts increases, the heterogeneity becomes more severe (in order of SSM3 > SSM2 > SSM1 > No-SS). As shown in (d), SSM3 exhibits the highest level of heterogeneity in the secondary cases (the highest frequency at zero and also the largest right tail). This is consistent with the results of the basic reproduction number, as shown in Figure 7.

**Figure 11:**
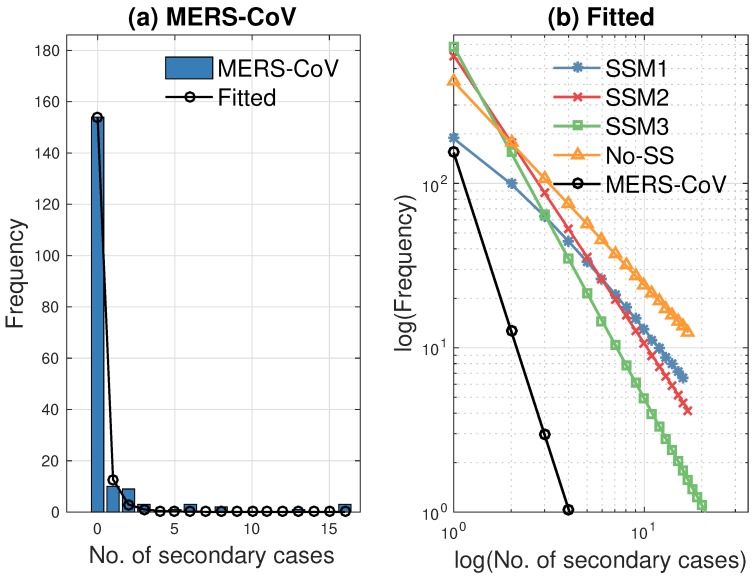
(**a**) Distribution of secondary cases for the 2015 Korean MERS-CoV data and its fitted power law distribution are illustrated. (**b**) Four fitted power law distributions using λ=10% are compared with MERS-CoV data. SSM3 shows the best fit to the distribution of secondary cases of the MERS-CoV data (see the slopes of the squared and circled lines).

We have fitted the distribution with a power function $\left(ax^{b}\right)$, where *x* is the number of secondary cases, and compared the slope of each fitted function to the 2015 MERS-CoV data. First, the left panel in Figure 11 displays the distributions of secondary cases for the 2015 MERS-CoV (blue bar) and the fitted power law distributions (black circled curve). Next, the distributions of secondary cases are fitted as a power law using the results under four different models with λ=10%. The right panel compares four fitted power law distributions with the MERS-CoV data. Obviously, SSM3 shows the best fit to the distribution of secondary cases for the MERS-CoV data (compare the slopes of the green squared and black circled lines). Parameters (*a* and *b* in $ax^{b}$) are estimated for the MERS-CoV data and the four models by the least-squares method. For the least-squares fitting procedure, we used the Levenberg—Marquardt method with line-search implemented in MATLAB (The Mathworks, Inc., Natick, MA, USA) in the built-in routine *lsqcurvefit* which is part of the optimization toolbox. The resulting parameter estimates are listed in Table 3.

### 3.5. The Impact of Isolation Intervention Strategies

**Figure 12:**
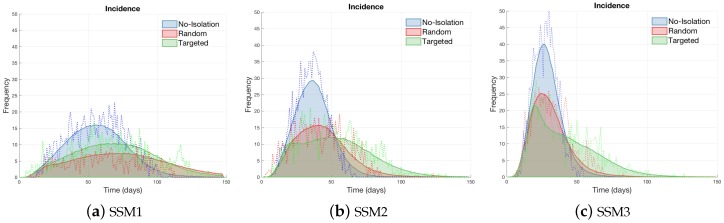
The impacts of two isolation strategies on the average number of MERS-CoV cases are compared without isolation ($\beta_{0}$ = 0.7 is used). Random selection reduces incidence proportionally while targeted isolation reduces the peak size and delay the peak time.

Lastly, we investigate the impact of isolation interventions on the MERS-CoV dynamics. Two distinct isolation strategies are implemented: a random and a targeted intervention. First, a random intervention isolates individuals (20% randomly chosen; both regular and super-spreaders) from the S and E classes at t=14 day. Second, a targeted intervention isolates individuals (super-spreaders only starting from t=14 day until the end of epidemic duration) from the E and I classes. Here, a targeted isolation intervention means an individual is isolated and either has effective contact with infected individuals (exposed) or is infected by super-spreaders. The results under the three models (SSM1, SSM2 and SSM3) are displayed in Figure 12. In all three models, a random selection reduces the peak size proportionally, resulting in a longer epidemic duration (red incidence curves in (a), (b), (c)).

Please note that a targeted isolation reduces the peak size significantly at the beginning which can delay the highest peak and result in more preparation time (green incidence curves in (b), (c)). The implementation of targeted isolation while incapable of eliminating a possible outbreak, still manages to reduce the magnitude of the epidemic peak by distributing both the infection cases and hospitalization over a broader window of time. Distributing the MERS-CoV case burden over long windows in time is highly desirable when resources are limited.

A targeted intervention is more effective in both SSM2 and SSM3, while a random intervention is more effective in SSM1. It is worth noting that the effectiveness of interventions depends on the infected-network structures. These results suggest that the effectiveness of interventions depends on the characteristics of the MERS-CoV transmission dynamics. Hence, it highlights the roles of super-spreaders in the transmission dynamics of infectious disease and planning future interventions.

## 4. Discussions

In this study, we built a stochastic agent-based model on the 2015 MERS-CoV outbreak in Korea from the perspective of SSE. The outbreak was an SSE: almost 80% of 186 confirmed cases were infected by five super-spreaders (147/186). Surprisingly, one super-spreader alone infected 53% of the MERS-CoV cases via other super-spreaders (79/147). To devise effective intervention strategies and countermeasures for future emerging infectious diseases, it is necessary to clarify the generating mechanism of SSEs. In this regard, agent-based modeling is a useful tool for incorporating individual heterogeneity into the epidemic model. The present research developed and analyzed agent-based models with different assumptions concerning the feature of super-spreaders.

Our results indicate that the connectivity of individuals and a higher level of infectivity are the most significant factors for the MERS-CoV transmission dynamics. The effect of super-spreaders was not remarkably significant in SSM1, but it was in SSM2 and SSM3. The higher baseline transmission rate and the longer contact range produced a more rapid and severe disease spread in SSM2 and SSM3 than in SSM1. Due to the features of super-spreaders, the real-world data demonstrated the best fit with SSM3 compared to SSM1 or SSM2. The impact of the effective radius on the basic reproduction number $R_{0}$ was the most significant. Also, higher transmission rates led to the larger $R_{0}$.

Furthermore, the impact of super-spreaders on the distributions of $R_{0}$ under SSM3 became more significant as a proportion of super-spreaders increased (the largest right tails in SSM3), which was consistent with the distributions results of secondary cases. SSM3 showed the highest level of heterogeneity in the distributions of $R_{0}$ and secondary cases while No-SS gave the least heterogeneity. All results above suggest that SSE can be more properly characterized by a small number of infectious individuals who can infect others by means of a larger amount of contact with others combined with higher infectivity. Having only one feature (either higher infectivity or higher connectivity) is not strong enough to generate such a high level of heterogeneity in secondary cases.

The effectiveness of two isolation strategies was examined: a random isolation reduced incidence proportionally while a targeted isolation reduced the peak size and delayed the peak time. A targeted isolation was more effective in both SSM2 and SSM3 while a random isolation was more effective in SSM1. These results suggest that the effectiveness of intervention strategies is dependent on the structure of the infection network. However, super-spreaders can be identified in retrospectively; it is very challenging to predict SSEs or super-spreaders. Thus, a targeted isolation intervention might be idealistic to be implemented. Nevertheless, we can investigate the most critical factors for generating SSEs and these factors can provide how to devise effective intervention strategies as discussed in [5,22]. For instance, various factors include co-infection with another pathogen, immune suppression, changes in airflow dynamics, delayed hospital admission, misdiagnosis, and inter-hospital transfers. These factors are worthy of investigation and should be incorporated into targeted intervention strategies. Our results showed that even when early detection was unsuccessful, it is still effective to find possible candidates for super-spreaders and isolate them to reduce potential risks of severe outbreaks.

One of the limitations of this research lies in the shape of the space where the simulations were executed. Although it is well known that infectious disease transmission dynamics depends on the structure of the infection network, we based our model on a random network in order to focus on the effect of super-spreaders on disease transmission. Despite this simplification, our results from a simple random network clearly demonstrated the role of super-spreaders on disease transmission and the possible effective intervention scenarios. Further studies are expected to conduct epidemic models based on other network structures, especially more realistic ones. For example, using the empirical contact network of the 2015 MERS-CoV transmission in South Korea would make the simulated space more realistic. Also, the effectiveness of more various intervention strategies and possible epidemic scenarios can be explored in future research.

## 5. Conclusions

We developed an agent-based model that can provide a general mathematical tool for any disease outbreaks with super-spreading events. Super-spreading events in the transmission dynamics of 2015 MERS-CoV showed a high level of heterogeneity. Heterogeneity in transmission patterns are one of the most critical factors for infectious disease transmission dynamics. Our agent-based model has employed real MERS-CoV epidemic features based on the 2015 MERS-CoV epidemiological data. Our findings highlight the roles of super-spreaders in a high level of heterogeneity, underscoring that the number of contacts combined with a higher level of infectivity are the most critical factors for substantial heterogeneity in generating secondary cases of the 2015 MERS-CoV transmission. Having only one feature (either higher infectivity or higher connectivity) is not strong enough to generate such a high level of heterogeneity in secondary cases.

## Figures and Tables

**Table 1:** Baseline parameter values.

Parameter	Description	Value	Reference
$\beta_{0}$	Background transmission constant	1	Estimated
1/κ	Mean incubation period	9	[1]
$\sigma_{\kappa }$	Standard deviation of incubation period	16	[1]
1/γ	Mean infectious period	21	[1]
$\sigma_{\gamma }$	Standard deviation of infectious period	76	[1]
λ	Proportion of super-spreaders	0–0.1	[15]
$r_{0}$	Effective radius	$\sqrt{6}$	[15]
α	Exponent in transmission rates	2	[15]

**Table 2:** Mean (M) and standard deviation (σ).

λ	Epidemic Output	No-SS	SSM1	SSM2	SSM3
λ=4%	Peak time	M=59.57	M=51.34	M=41.26	M=34.57
		σ=21.0	σ=18.71	σ=14.77	σ=12.41
	Peak size	M=24.42	M=27.99	M=36.66	M=46.12
		σ=7.83	σ=9.49	σ=11.50	σ=14.06
	Epidemic duration	M=118.28	M=103.73	M=85.02	M=73.24
		σ=33.12	σ=31.55	σ=25.17	σ=21.21
λ=10%	Peak time	M=59.57	M=41.42	M=29.12	M=23.29
		σ=21.00	σ=15.72	σ=9.15	σ=7.90
	Peak size	M=24.42	M=33.86	M=53.70	M=69.02
		σ=7.83	σ=11.86	σ=14.98	σ=21.38
	Epidemic duration	M=118.28	M=87.86	M=61.69	M=48.16
		σ=33.12	σ=28.49	σ=17.31	σ=14.25

**Table 3:** Parameter estimation for the distributions of secondary cases.

	MERS-CoV	No-SS	SSM1	SSM2	SSM3
a	153.9127	430.2658	501.5440	542.4199	598.1410
b	−3.6030	−1.2061	−1.4711	−1.6836	−2.0776
